# Prolonged intermittent theta burst stimulation targeting the left prefrontal cortex and cerebellum does not affect executive functions in healthy individuals

**DOI:** 10.1038/s41598-024-61404-9

**Published:** 2024-05-24

**Authors:** Mei Xu, Stevan Nikolin, Adriano M. Moffa, Xiao Min Xu, Yon Su, Roger Li, Ho Fung Chan, Colleen K. Loo, Donel M. Martin

**Affiliations:** 1 Discipline of Psychiatry and Mental Health, Faculty of Medicine and Health, School of Clinical Medicine, University of New South Wales, High St, Kensington, Sydney, NSW 2052 Australia; 2 Black Dog Institute, Sydney, Australia; 3 The George Institute for Global Health, Sydney, Australia

**Keywords:** iTBS, ERP, DLPFC, Cerebellum, Cognition, Neuroscience, Psychology

## Abstract

Repetitive transcranial magnetic stimulation (rTMS) for alleviating negative symptoms and cognitive dysfunction in schizophrenia commonly targets the left dorsolateral prefrontal cortex (LDLPFC). However, the therapeutic effectiveness of rTMS at this site remains inconclusive and increasingly, studies are focusing on cerebellar rTMS. Recently, prolonged intermittent theta-burst stimulation (iTBS) has emerged as a rapid-acting form of rTMS with promising clinical benefits. This study explored the cognitive and neurophysiological effects of prolonged iTBS administered to the LDLPFC and cerebellum in a healthy cohort. 50 healthy participants took part in a cross-over study and received prolonged (1800 pulses) iTBS targeting the LDLPFC, cerebellar vermis, and sham iTBS. Mixed effects repeated measures models examined cognitive and event-related potentials (ERPs) from 2-back (P300, N200) and Stroop (N200, N450) tasks after stimulation. Exploratory non-parametric cluster-based permutation tests compared ERPs between conditions. There were no significant differences between conditions for behavioural and ERP outcomes on the 2-back and Stroop tasks. Exploratory cluster-based permutation tests of ERPs did not identify any significant differences between conditions. We did not find evidence that a single session of prolonged iTBS administered to either the LDLPFC or cerebellum could cause any cognitive or ERP changes compared to sham in a healthy sample.

## Introduction

Repetitive transcranial magnetic stimulation (rTMS) has demonstrated therapeutic potential for different neuropsychiatric conditions^1,2^, including potential cognitive enhancing effects^3–5^. Due to the current lack of effective treatments^6,7^, rTMS has additionally been explored as a novel intervention for the negative symptoms and cognitive deficits in patients with schizophrenia, with several clinical studies^8–10^ and meta-analyses^11–13^ suggesting promising therapeutic effects. RTMS involves the delivery of multiple magnetic pulses to target specific brain regions of interest for the purpose of modulating and causing long-lasting aftereffects on brain activity^1^. RTMS can be categorised into high-frequency rTMS (HF-rTMS: ≥ 5 Hz), typically producing cortical facilitation and excitatory effects^1,2^, and low-frequency rTMS (LF-rTMS: < 5 Hz), which typically induces inhibitory aftereffects^1^.

The left dorsolateral prefrontal cortex (LDLPFC) has been a common target for excitatory rTMS in clinical populations^12,14–16^ to examine for potential cognitive enhancing effects. Clinical trials and several meta-analyses and reviews^11,12,17^ have shown therapeutic effects of rTMS targeting the LDLPFC for negative symptoms and cognitive functioning in schizophrenia^18–21^. However, these findings have been contradicted by other meta-analytic^22^ and randomised controlled studies of excitatory rTMS targeting the LDLPFC, which have reported no effect compared to control conditions^23–25^. These metal-analytic studies have further found that clinical studies to date have implemented diverse rTMS protocols with variable stimulus parameters, including stimulus frequency (i.e., 5 Hz, 10 Hz, 20 Hz rTMS, iTBS), stimulus intensities (80–120% resting motor threshold, RMT), number of sessions (4–40 sessions), and total number of pulses (1200–80,000)^12^. The optimal effective stimulus parameters for treating negative symptoms thus remain to be determined.

Interestingly, emerging research has further indicated that excitatory rTMS targeting the cerebellum may have benefits for improving negative symptoms or cognitive impairment in schizophrenia^9,10,26^. Several clinical studies have provided evidence supporting the use of iTBS targeted over the cerebellar vermis to improve negative symptoms in schizophrenia^9,27^. Demirtas-Tatilidede and colleagues conducted the first open-label study of cerebellar iTBS in people with schizophrenia, delivering 10 sessions at 100% active motor threshold, finding an improvement in negative symptoms and cognition after rTMS^28^. Nevertheless, the broader literature shows mixed effects of iTBS on this site in people with schizophrenia^26^. So far, there have been four randomised controlled trials using standard iTBS targeting the cerebellum for negative symptoms^26^. However, there are several methodological differences in iTBS protocols across these cerebellar studies, including intensity (80%, 100% RMT), targeting approach (MRI guided, scalp measurement), and control condition (inactive sham, angle rotation) potentially contributed to the variation in outcomes among studies. Further, few prior studies have investigated the potential of prolonged iTBS for negative symptoms in this region.

Research into rTMS targeting the LDLPFC and cerebellum as treatments for schizophrenia's negative symptoms has shown promising potential^11,12^. However, an optimal rTMS protocol for these symptoms and related cognitive impairments remains elusive, hindered by the disorder's varied presentation, cortical anomalies, and the effects of concurrent medication impacting TMS effects^29^. In this context, following a well-established translational approach, preclinical experimental studies conducted in healthy participants have potential utility for informing on the relative effects of different stimulation parameters and TMS target sites, with the benefits of minimising confounders and the burden of cost. This approach is intended to refine stimulation techniques and assess the cognitive benefits of rTMS in healthy populations, with the aim of extending these findings to schizophrenia and other relevant psychiatric conditions.

In healthy brains, the DLPFC has been a pivotal target site for rTMS to examine potential cognitive enhancing effects^3,30^. Several rTMS studies and reviews have confirmed the beneficial effects of targeting the DLPFC with an excitatory rTMS session on higher cognitive functions^3,21,30–33^. For example, in our recent meta-analysis, which included the majority of studies targeting the DLPFC, we showed that excitatory rTMS significantly improved accuracy and reaction times on measures of executive functions, including cognitive control and working memory^30^. This meta-analysis showed large variability between studies for excitatory rTMS stimulus parameters (pulse frequencies: 5 Hz, 6 Hz, 10 Hz, 20 Hz, 25 Hz, iTBS; stimulus intensities: 70–120% RMT, number of pulses per session:300–1800 and number of sessions: 1–10) and identified that the optimal stimulus parameters remained to be determined. Prolonged iTBS targeting DLPFC has emerged to modulate cortical excitability and enhance cognition with stronger cumulative effects compared to the conventional iTBS^34^. To investigate the neural mechanisms underlying the cognitive effects of rTMS, several prior studies have used electroencephalography (EEG) to measure neurophysiological effects from stimulation of this region and shown positive EEG effects^31,32,35^ even in the absence of significant changes in behavioural outcome measures^36^.

Traditionally, the function of the cerebellum, in contrast, has been considered to be associated with motor control; however, neuroimaging studies have provided evidence of its involvement in cognitive processes, including attention, language, memory and executive functions^37–40^. Specifically, the fronto-cerebellar loop has been implicated in subserving working memory^39^ and cognitive control^41,42^ functioning. Recent research using iTBS has further implicated the role of the cerebellar vermis in higher-level cognitive functions through cerebello-cerebral circuits^38,43,44^. Yet, there are very limited studies examining the cognitive effects of excitatory rTMS over the cerebellum for improving cognition^38^. To the best of our knowledge, no prior study has explored the relative cognitive or neurophysiological correlates of excitatory rTMS administered to both the LDLPFC and cerebellar vermis in healthy participants.

To provide further insights into whether excitatory TMS targeting the LDLPFC or cerebellum vermis may be more promising for improving cognition, this study aimed to better understand the neurophysiological effects of rTMS to these two brain sites by investigating its effects on higher level neurocognitive function and electrophysiological measures, in healthy participants. We investigated the effects of prolonged iTBS, which has recently emerged as a promising new type of rTMS for improving clinical symptoms and cognition^45,46^. We hypothesised that: prolonged iTBS administered over the LDLPFC and cerebellar vermis would produce better behavioural outcomes in working memory and cognitive control compared to sham; and that prolonged iTBS targeting LDLPFC and cerebellum would significantly improve brain activity measures assessed with event-related potentials (ERPs) on EEG associated with working memory and response inhibition compared to sham.

## Materials and methods

### Participants

The study was powered to detect at least a moderate sized difference (d = 0.5) between the two active rTMS conditions and sham rTMS with a sample size of N = 40 (alpha = 0.05, power = 0.8). Fifty healthy participants were recruited in this study allowing for an expected dropout, missing or incomplete data of 20%. Inclusion criteria: individuals aged between 18 and 40 years; right-handed (assessed using the Edinburgh handedness test); not taking any concurrent medications which may affect brain stimulation effects (e.g., benzodiazepines, pseudoephedrine, psychotropics); no current history of drug or alcohol abuse or dependence; not a smoker (exclude the impact of chronic smoking on cortical excitability)^47^; no current self-reported neurological or psychiatric disorders; no recent head injury (in the last three months)^48^; no history of seizure, stroke, or any serious medical conditions; not currently pregnant. All participants were assessed using the ‘Transcranial Magnetic Stimulation Adult Safety Screen (TASS)’ to ensure no risk factors for receiving TMS^49^. All participants provided written informed consent to participation and the experimental protocol was approved by the UNSW Human Research Ethics Committee (HC210639) following the Code of Ethics of the World Medical Association (Declaration of Helsinki)^50^.

### Procedure

**Figure 1:**
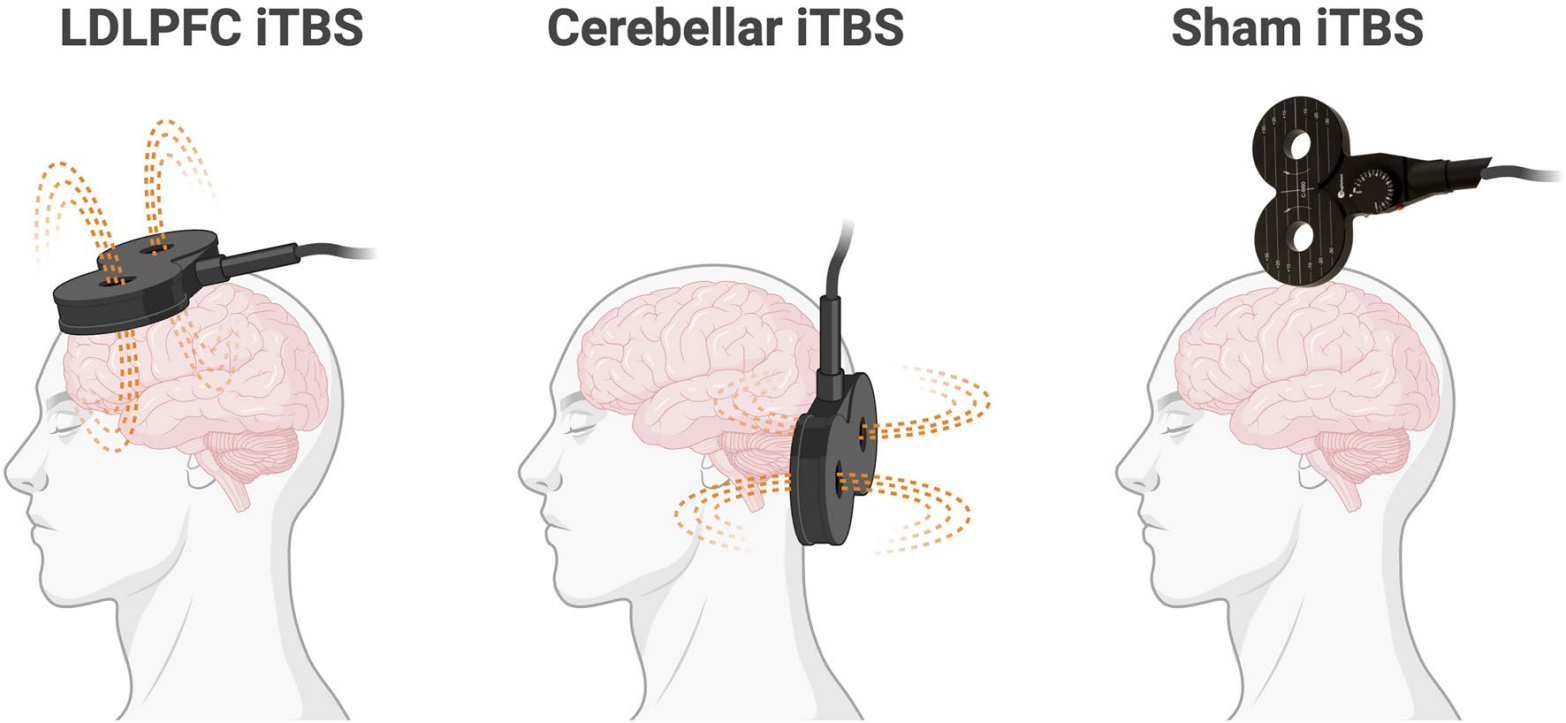
iTBS administrations. *LDLPFC* left dorsolateral prefrontal cortex, *iTBS* intermittent theta burst stimulation. Sham iTBS targeted the vertex (Cz of the 10–20 EEG system) with a stimulation intensity of 15% of the maximum stimulator output.

**Figure 2:**
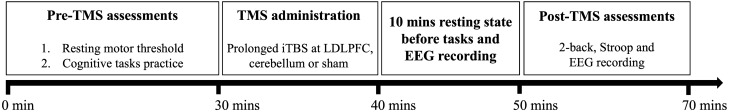
Experimental procedure. *TMS* transcranial magnetic stimulation, *LDLPFC* left dorsolateral prefrontal cortex, *iTBS* intermittent theta burst stimulation, *EEG* electroencephalography.

This study used a randomised single-blind cross-over study design. Only participants were blinded to the assignment. The TMS administrator and EEG researchers were not blinded to the different TMS conditions. All participants underwent three iTBS sessions: a single session of LDLPFC iTBS, a single session of cerebellar iTBS, and a single session of sham iTBS (see Fig. 1). Each session was conducted at least 48 hours apart. Following the administration of active or sham iTBS, there was a 10 mins rest period^36,51^. Participants were asked to practise cognitive tasks before TMS stimulation was administrated in each session. After a single session of iTBS, participants completed the 2-back working memory task and Stroop task. The sequence of sessions and tasks was determined through a pseudorandomised and counterbalanced approach using a computer-generated randomisation table in R across all participants. Electrophysiological outcomes were assessed 10 min after stimulation and recorded approximately 11 mins of 2-back working memory task and 10.5 mins of Stroop task respectively (see Fig. 2).

### Transcranial magnetic stimulation (TMS)

iTBS was developed to mimic the brain's intrinsic long-term potentiation (LTP) firing patterns, marking its role as a quick-acting excitatory rTMS method^52^. Standard iTBS treatment, lasting around 3 mins with a total of 600 pulses, is recognised for its comparable effectiveness to a 10 Hz rTMS session administered over approximately 40 mins, in the treatment for treatment-resistant depression^53^. The advent of prolonged iTBS protocols, extending the stimulation to approximately 9 mins with 1800 pulses, has revealed its capability for rapid antidepressant effects in individuals with treatment-resistant depression^45,46^ and for enhancing executive cognitive functions in healthy subjects^34,54^. The current research incorporates this prolonged iTBS approach to further explore its potential impacts.

Participants were randomly assigned to receive 9 mins and 40 s of prolonged LDLPFC, cerebellar and sham iTBS (1800 pulses per session). Recent clinical studies have suggested that an accelerated treatment course of iTBS has potent clinical effects^26,45,46^ and beneficial effects on cognition^34^. The RMT was measured and determined as the lowest machine output in which 50% of the delivered stimuli (three out of six trials) produced a peak-to-peak motor-evoked potential of at least 50 uV. 80% RMT was used in prolonged LDLPFC and cerebellar iTBS sessions. TMS was delivered using a MagVenture MagPro X100 system (MagVenture A/S, Denmark) equipped with a double-sided MagVenture Cool-B65 coil for LDLPFC and sham iTBS, and a double-cone MagVenture Cool-B60 coil for cerebellar iTBS. Computational modelling conducted using SimNIBS (3.2.6) showed that this approach led to approximately equivalent electric fields at the respective brain regions of interest. A neuronavigation system (Xensor, eemagine Medical Imaging Solutions GmbH, Berlin, Germany) was used to accurately position the TMS coil over the individualised stimulation target for each session using a default average head model as a standard reference. LDLPFC iTBS was localised to MNI coordinate (− 38, 44, 26)^55^. The cerebellar vermis was targeted at MNI coordinate (1, − 73, − 33)^10,56^. For sham iTBS, the coil was placed perpendicularly to the vertex (Cz using the 10–20 EEG system) with the edge of the coil touching the head and stimulation (see Fig. 1) was delivered at 15% of the maximum stimulator output^57,58^, to mimic somatosensory sensations.

### Behavioural measures

#### 2-back

After prolonged iTBS, participants were asked to perform a visual 2-back working memory task programmed using Inquisit software (Version 5, Millisecond Software). Neuroimage studies have demonstrated brain activation in the prefrontal cortex and cerebellum during n-back performance^39^. In the 2-back task, a sequence of letters ranging from B to J was presented on screen for 100 ms with an interstimulus interval of 1900 ms between successive letters. The task was presented for approximately 11 mins, including three blocks with a total of 336 stimulus comprising 25% target trials and 75% distractor trials. Participants were instructed to respond whenever the current letter matched a letter observed in two trials previously (letter ‘M’ on a keyboard), and when the current letter did not match (letter ‘N’ on a keyboard). Participants were given the opportunity to have a two-minute practice block before the formal experiment started. This was repeated as necessary to ensure that participants understood the task instructions. Task performance was assessed using response time (RT) for correct target responses and d-prime^59^, a measure of discriminative sensitivity.

#### Stroop

The Stroop task was performed after prolonged iTBS for approximately 10.5 mins using Inquisit software (Version 5, Millisecond Software). Neuroimaging studies have suggested the involvement of fronto-cerebellar connectivity during Stroop performance^41,42^. Participants were seated in a chair facing a monitor. The words ‘RED’, ‘GREEN’, ‘BLUE’ or ‘YELLOW’ were presented on a black screen either as congruent trials using the same colour as the word meaning (e.g., the word ‘RED’ in red), or as incongruent trials using a different colour to the word meaning (e.g., the word ‘RED’ in green). Each stimulus was displayed for 1000 ms with a 1000 ms interstimulus interval before the next stimulus appeared. Half of the words were congruent trials (156 stimulus), while the other half were incongruent trials (156 stimulus). Participants were instructed to use the index and middle fingers of both hands to press ‘F’ (red), ‘G’ (green), ‘J’ (blue), and ‘K’ (yellow) on the keyboard in response to the colour of words as fast and accurately as possible while ignoring the semantic meaning of the stimuli. This task consisted of three blocks with each block containing 104 stimulus (312 trials in total). Participants had the opportunity to practice the Stroop task to fully understand the instructions. Task performance was assessed separately for congruent and incongruent trials using response times for correct responses.

### Electroencephalography (EEG) acquisition

A TMSi Refa amplifier (TMS International, Oldenzaal, Netherlands) with a 64-channel setup was used to collect EEG data at a sampling rate of 2048 Hz. The processing and analysis of EEG data were carried out using the Fieldtrip open-source toolbox^60^ with custom MATLAB scripts (v.R2022a; MathWorks). All scripts used for EEG processing can be accessed through the following link (https://github.com/EchoXu9/LDPFC-Cerebellum-iTBS). Initially, a Butterworth IIR digital filter was used to eliminate 50 Hz line noise. Subsequently, a second-order band-pass filter was applied, with cut-off frequencies set at 0.05 and 70 Hz. Cognitive task data were segmented into trials, starting 0.5 s before the onset of each stimulus and extending for 1.5 s after the stimulus presentation. Following this, a manual procedure was employed to identify and reject trials and channels with significant artefacts. Independent Components Analysis (ICA) was used to remove non-cortical physiological activity (e.g., cardiac, muscle, ocular) and non-physiological activity (e.g., environmental noise, movement). This ICA was conducted using the default *runica* function implementation within Fieldtrip toolbox. Finally, the data was re-referenced using the common average reference.

### Event-related potentials (ERPs)

ERP components of interest include P300 and N200 for the n-back task^61–63^, and N200 and N450 for the Stroop task^63,64^. These were selected as prior sham-controlled excitatory rTMS studies detected changes in these components during the n-back task^31,35^ and the Stroop task^32^. A collapsed localiser approach^65^ was adopted to compute grand-average ERPs (P300 and N200 for the 2-back task; N200 and N450 for the Stroop task) across all participants and sessions. We used the resultant topographical representations to locate a sample-specific region of interest for each ERP component. Subsequently, the resultant waveform plot from each participant with the three sessions collapsed was used to select the time interval for individualised ERP component amplitudes across all participants.

For ERPs of the 2-back task (i.e., P300, N200), we calculated averages from target hit trials; while for ERPs of the Stroop task (i.e., N200, N450), averages were computed separately for congruent and incongruent trials. The mean amplitudes for P300, N200 and N450 were extracted from parietal EEG channel Pz, frontal EEG channel Fz and central EEG channel Cz, respectively. To establish the appropriate time windows for analysis, we identified time windows by computing the participant average ERP components with three sessions collapsed and visually inspected the mean latency for the early ERP components: N200 and P300 from 2-back and Stroop tasks. For N450 of the Stroop task, the time window containing the largest power difference between congruent and incongruent trials was selected by visual inspection, and the latency of N450 was selected at 475 ms post-stimulus (time window: 425–525 ms).

### Statistical analysis

For the primary analyses, mixed effects repeated measures models (MRMMs) were performed using the ‘lme4’ package in R (version 4.3.0). MRMMs for behavioural data (RT, D-prime from 2-back; RT from Stroop) and ERPs outcomes (P300, N200 from 2-back; N200, N450 from Stroop) included the fixed effects of ‘Condition’ (LDLPFC iTBS, Cerebellar iTBS and Sham iTBS) and ‘Session’ (Session 1, 2 and 3) with a random effect of ‘Participant’. As demographic factors did not significantly improve the fit of the MRMMs, we did not include these factors in the final models. Additionally, we conducted non-parametric cluster-based permutation tests (two-tailed) using MATLAB (v.R2022a; MathWorks) on ERP data as an exploratory measure. The cluster-based permutation tests were identified as instances where two or more consecutive time samples or adjacent EEG channels exhibited a p-value < 0.05 within a dependent sample repeated-measures ANOVA^66^. The permutation test controls for the family-wise error rate while performing statistical comparisons across a large spatiotemporal parameter space. Trials were randomly permuted in 3000 iterations, using ERP data from all EEG channels within the time interval 0–700 ms from stimulus onset. Significant clusters required at least one neighbouring channel. We selected at least one neighbouring channel as a less conservative threshold to explore further possibilities^67^. All analyses are consistent with the OSF preregistration^68^. Reaction times of trials in cognitive tasks that were too fast (< 200 ms) or slow (2-back: > 2000 ms, Stroop: > 1000 ms) and sessions with an error rate greater than 50% were not included in further analyses. A significance level with *p* value < 0.05 was used to determine statistical significance. Effect sizes of all behavioural and ERP outcome measures using Cohen d were reported. We used the ‘Durga’ package in R, ‘Fieldtrip’ and ‘UnivarScatter’ toolboxes in MATLAB to visualise the data.

## Results

### Demographic characteristics

Fifty participants were recruited and randomly assigned to different conditions. A total of 43 participants completed all three sessions, of which 42 participants were included for analyses for the behavioural and EEG data in the 2-back task (age: 25.6 ± 5.3 years; gender: 17 M and 25 F; education: 17.3 ± 3.0 years) and 43 participants for the Stroop task (age: 25.5 ± 5.4 years; gender: 17 M and 26 F; education: 17.3 ± 3.0 years). Six participants withdrew from the study (DLPFC: 4; Cerebellum: 2) due to adverse events associated with iTBS stimulation., and one additional participant withdrew due to acute anxiety that was unrelated to the study (sham condition). One participant was excluded from analyses on the 2-back task as this participant did not fully understand the instructions of this task resulting in invalid responses for the majority of trials. Statistical analyses using MRMMs can handle missing data as it is assumed to be missing at random.

### Behavioural outcomes

There were no significant differences between conditions for 2-back task response times (cerebellum vs. sham: *d* = -0.16; LDLPFC vs. sham: *d* = 0.13; LDLPFC vs. cerebellum: *d* = 0.28; see Table 1 and Fig. S1) and D-prime (cerebellum vs. sham: *d* = − 0.15; LDLPFC vs. sham: *d* = 0.09; LDLPFC vs. cerebellum: *d* = 0.23; see Table 1 and Fig. S1).

**Table 1:** Summary of results for behavioural outcomes in 2-back and Stroop.

Condition	Estimate	SE	z-value	*d*	95% CI LB	95% CI UB	*p* value
2-back RT (ms)							
Sham	723	24	30.04	–	676	771	–
Cerebellum	714	24	29.17	0.16	666	762	0.43
DLPFC	736	24	30.33	− 0.13	689	784	0.27
2-back D-prime							
Sham	1.93	0.14	13.93	–	1.66	2.20	–
Cerebellum	1.80	0.14	12.70	0.15	1.52	2.07	0.09
DLPFC	1.97	0.14	14.04	− 0.09	1.69	2.24	0.64
Stroop RT congruent trials (ms)							
Sham	584	10	60.11	–	565	603	–
Cerebellum	590	10	59.99	0.10	571	610	0.18
DLPFC	590	10	60.16	0.11	572	610	0.16
Stroop RT incongruent trials (ms)							
Sham	633	12	52.70	–	609	657	–
Cerebellum	635	12	52.30	0.04	612	659	0.63
DLPFC	637	12	52.57	0.00	613	661	0.42

*SE* standard error, *CI* confidence interval, *LB* lower bound, *UB* upper bound, *RT* reaction time, *DLPFC* dorsolateral prefrontal cortex. For DLPFC and cerebellar stimulations, *p* values were computed compared to sham. Two-tailed tests and a significance level with *p* value < 0.05 were used.

Likewise, there were no significant differences between conditions for reaction times on the Stroop task for congruent trials (cerebellum vs. sham: *d* = 0.10; LDLPFC vs. sham: *d* = 0.11; LDLPFC vs. cerebellum: *d* = 0.02; see Table 1 and Fig. S1) or incongruent trials (cerebellum vs sham: *d* = 0.05; LDLPFC vs. sham: *d* = 0.00; LDLPFC vs. cerebellum: *d* = 0.05; see Table 1 and Fig. S1).

### Event-related potentials (ERPs)

**Figure 3:**
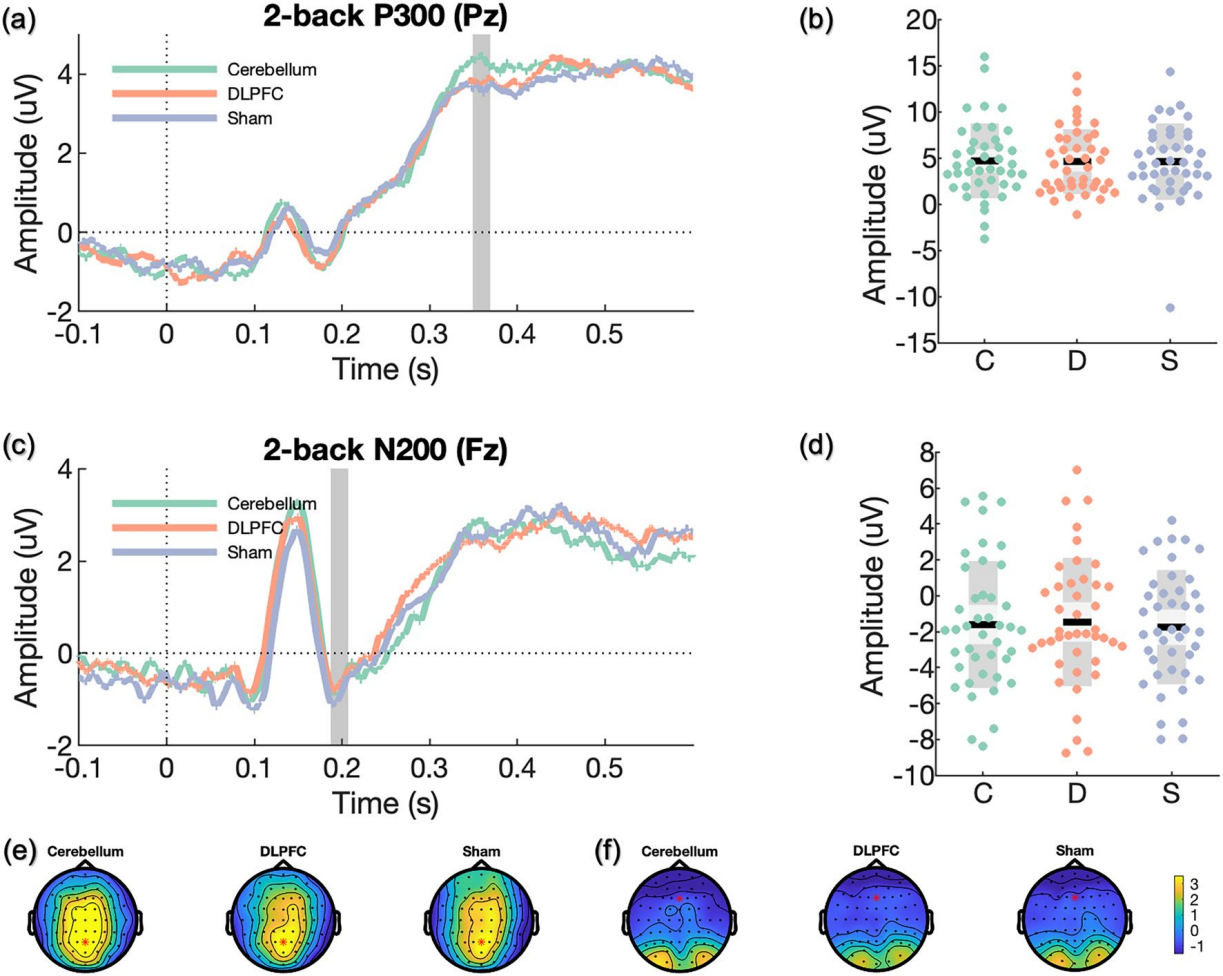
Event-related potentials (ERPs) during the 2-back working memory task. In scatter plots, black lines show the mean, light grey shaded boxes indicate the standard deviation, dark grey regions indicate the 95% confidence interval, *C* cerebellar iTBS, *D* LDLPFC iTBS, and *S* sham iTBS. ERP plots were corrected at baseline from − 0.5 to 0 s, and the downward baseline trend was likely due to anticipatory effect before the task. (**a**) P300 were selected at a midline parietal channel (Pz) following the targets’s correct trials on the 2-back task. The grey region indicates the time window using the grand average approach. (**b**) Scatter plot of P300 component. The amplitude of P300 was extracted at Pz based on individualised time windows for all participants. (**c**) N200 was inspected at a midline frontal channel (Fz) following target correct trials on the 2-back task. The grey region indicates the time window following the grand average approach. (**d**) Scatter plot of N200 component. The amplitude of N200 was extracted at Fz based on individualised time windows for all participants. (**e**) Topographies of the P300 component for all three conditions after prolonged iTBS (359 ± 20 ms). (**f**) Topographies of the N200 component for all three conditions after prolonged iTBS (197 ± 20 ms).

Mixed effects models of 2-back task ERP outcomes did not show any significant differences between conditions for P300 at Pz (cerebellum vs. sham: *d* = 0.02; LDLPFC vs. sham: *d* = 0.00; LDLPFC vs. cerebellum: *d* = − 0.02; see Table S1 and Fig. 3) or N200 at Fz (cerebellum vs. sham: *d* = 0.05; LDLPFC vs. sham: *d* = 0.09; LDLPFC vs. cerebellum: *d* = 0.04; see Table S1 and Fig. 3).

**Figure 4:**
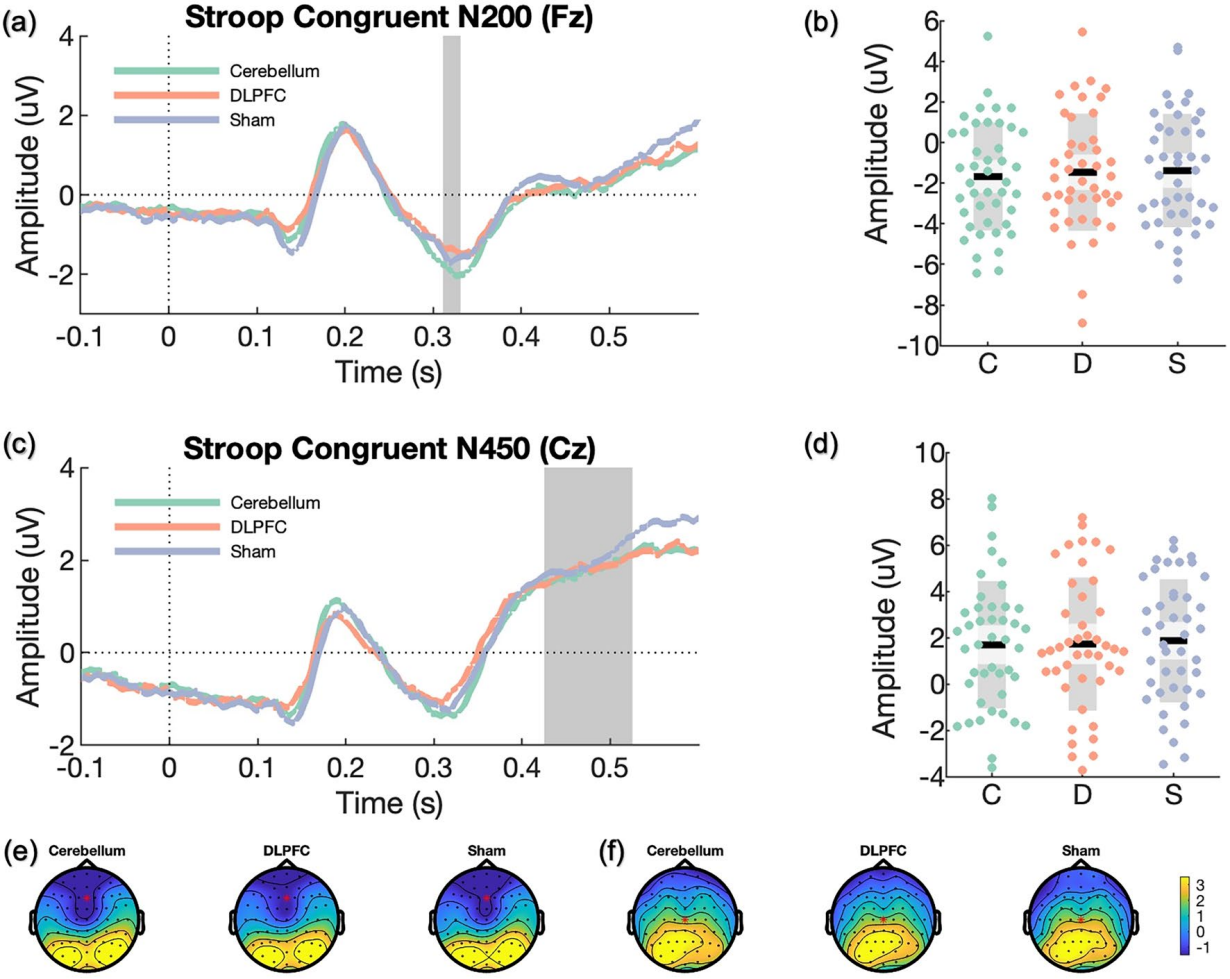
Event-related potentials (ERPs) of congruent trials during the Stroop task. In scatter plots, *C* cerebellar iTBS, *D* LDLPFC iTBS, and *S* sham iTBS. Black lines represent the mean, light grey boxes show the standard deviation, and dark grey regions indicate the 95% confidence interval. ERP plots were corrected at baseline from − 0.5 to 0 s, and the downward baseline trend was likely due to the anticipatory effect before the task. (**a**) N200 was selected at a midline frontal channel (Fz) following congruent correct trials on the Stroop task. The grey region indicates the time window of the grand average approach. (**b**) Scatter plot of N200 component. The amplitude of N200 was extracted at Fz based on individualised time windows for all participants. (**c**) N450 was inspected at a midline central channel (Cz) following congruent correct trials on the Stroop task. The grey region indicates the time window following the grand average approach. (**d**) Scatter plot of N450 component. The amplitude of N450 was extracted at Cz based on the grand average time window (475 ± 50 ms). (**e**) Topographies of the N200 component for all three conditions after prolonged iTBS (321 ± 20 ms). (**f**) Topographies of the N450 component for all three conditions after prolonged iTBS (475 ± 50 ms).

**Figure 5:**
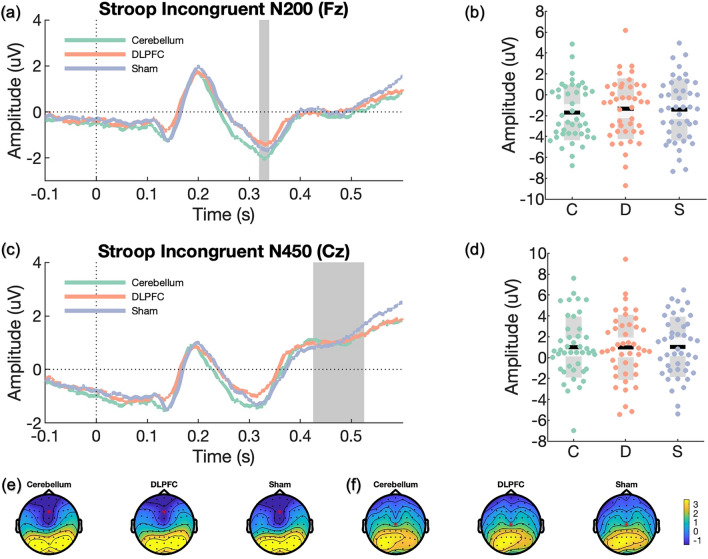
Event-related potentials (ERPs) of incongruent trials during the Stroop task. In scatter plots, *C* cerebellar iTBS, *D* LDLPFC iTBS, and *S* sham iTBS. Black lines represent the mean, light grey boxes show the standard deviation, and dark grey regions indicate the 95% confidence interval. ERP plots were corrected at baseline from − 0.5 to 0 s, and the downward baseline trend was likely due to anticipatory effect before the task. (**a**) N200 was selected at a midline frontal channel (Fz) following incongruent correct trials on the Stroop task. The grey region indicates the time window of the grand average approach. (**b**) Scatter plot of N200 component. The amplitude of N200 was extracted at Fz based on individualised time windows for all participants. (**c**) N450 was inspected at a midline central channel (Cz) following incongruent correct trials on the Stroop task. The grey region indicates the time window following the grand average approach. (**d**) Scatter plot of N450 component. The amplitude of N450 was extracted at Cz based on the grand average time window (475 ± 50 ms). (**e**) Topographies of the N200 component for all three conditions after prolonged iTBS (329 ± 20 ms). (**f**) Topographies of the N450 component for all three conditions after prolonged iTBS (475 ± 50 ms).

For the Stroop task, there were no significant differences between conditions for congruent ERPs, including N200 at Fz (cerebellum vs. sham: *d* = -0.14; LDLPFC vs. sham: *d* = − 0.04; LDLPFC vs. cerebellum: *d* = 0.09; see Table S1 and Fig. 4) and N450 at Cz (cerebellum vs. sham: *d* = − 0.10; LDLPFC vs. sham: *d* = − 0.07; LDLPFC vs. cerebellum: *d* = 0.02; see Table S1 and Fig. 4). Similarly, there were no significant effects for incongruent ERPs in N200 at Fz (cerebellum vs. sham: *d* = − 0.13; LDLPFC vs. sham: *d* = 0.04; LDLPFC vs. cerebellum: *d* = 0.16; see Table S1 and Fig. 5) and N450 at Cz (cerebellum vs. sham: *d* = − 0.01; LDLPFC vs. sham:* d* = − 0.03; LDLPFC vs. cerebellum: *d* = 0.02; see Table S1 and Fig. 5).

Exploratory cluster-based permutation testing also did not reveal any significant differences between conditions for ERP outcomes during 2-back and Stroop tasks.

### Blinding assessment and adverse effects

At the end of the last session, most participants (34/43, 79%) correctly guessed the sham condition with a confidence rate at 64% ± 28%. Three participants (3/50, 6%) reported strong headaches or scalp pain during or after prolonged iTBS. 12/50 (24%) participants reported mild or moderate discomfort (5/50, 10%), fatigue (5/50, 10%), headache (4/50, 8%).

## Discussion

To provide further insight into the cognitive effects of excitatory rTMS, we, therefore, conducted the current study to explore the relative cognitive and electrophysiological effects of LDLPFC and cerebellar prolonged iTBS compared to sham in healthy participants. Contrary to our hypotheses, we did not find any evidence for cognitive benefits or ERP effects during working memory or cognitive control with active rTMS administered to either brain region.

Despite a large sample size and a randomised sham-controlled cross-over design, we did not find evidence that prolonged iTBS over the LDLPFC induced behavioural or neuropsychological effects on working memory and cognitive control. This finding is in contrast with other studies that have reported significant behavioural effects with active rTMS targeting LDLPFC on such measures^31,33,69,70^. It is possible that methodological differences between studies (e.g., rTMS parameters, EEG and ERP processing methods) may explain discrepancies in findings. Notwithstanding, similar negative effects have been reported in other comparable studies investigating the offline cognitive effects of excitatory rTMS targeting the LDLPFC on working memory^35,54,70–72^ and the Stroop task^73,74^. For example, two recent prolonged iTBS (1800 and 1620 pulses per session, respectively) studies targeting the LDLPFC did not find RT and accuracy changes in the 2-back task compared to sham in healthy cohorts^54,72^. Likewise, Chung and colleagues conducted a TMS-EEG study and did not find significant changes in ERPs during the 2-back working memory task after a standard iTBS session and a prolonged iTBS (1200 pulses) session^35^. Similarly, recent studies which have investigated the effects of a single session of 10 Hz rTMS over LDLPFC showed no effect on the interference effect and behavioural responses in the Stroop task^73,74^. Taken together, these mixed findings suggest that a single session of prolonged iTBS has minimal behavioural or neurophysiological effects on working memory or cognitive control outcomes in healthy samples.

To the best of our knowledge, this is the first study to investigate the potential cognitive effects of a single session of prolonged cerebellar iTBS on working memory or cognitive control outcomes. The rationale was based on prior studies using rTMS which have implicated the cerebellar vermis in higher-order cognitive functions^43,44,56^. Results, however, showed that a single session of prolonged iTBS over the cerebellar vermis had no detectable effect on working memory or cognitive control behavioural or neurophysiological outcomes as measured with ERPs. This contrasts with prior rTMS studies which have reported positive neurophysiological effects following stimulation of this region^43,56^. For example, Esterman and colleagues^43^ utilised iTBS to target the region of the cerebellar vermis that activated as part of the dorsal attention network during an individualised resting-state fMRI and showed enhancement in attentional control compared to iTBS administered at the lateral cerebellum. The individualised neuronavigational approach is likely to produce superior cognitive effects of excitatory rTMS^75^. Taken together, there is, therefore, limited evidence that excitatory rTMS administered to the cerebellar vermis can modulate behaviour or EEG outcomes in healthy participants.

### Strengths and limitations

This study investigated, for the first time, the cognitive effects of prolonged iTBS administered to the LDLPFC and cerebellar vermis on working memory and cognitive control. Strengths of this study included: a relatively large-sized healthy sample, inclusion of ERP markers, and novel investigation of cerebellar vermis on cognitive and electrophysiological effects.

Potential limitations of the study include: (1) We used standardised MNI coordinates and an average head model to localise both targets, instead of individualised targets based on structural MRI, which potentially may have limited induced effects; (2) A subthreshold intensity of 80% RMT was administered for prolonged iTBS, which may have been insufficient for producing rTMS cognitive or effects on ERPs. However, we note that this intensity is similar to other HF-rTMS studies investigating cognitive enhancement in healthy participants (i.e., < 100% RMT)^30^ and no significant EEG difference was detected between suprathreshold (120% RMT) and subthreshold (80% RMT) of TMS over LDLPFC and cerebellum^76^; (3) Participants only received a single session of prolonged iTBS. It is possible that multiple rTMS stimulation sessions may produce more robust behavioural and neurophysiological effects; (4) Lastly, a significant proportion of participants (79%) were able to discern the sham condition, suggesting the possibility of compromised blinding. Nevertheless, there was no significant difference in any outcomes between sham and active conditions. Thus, blinding was not a critical factor in interpreting results. Notwithstanding, future studies should attempt to implement more convincing rTMS blinding conditions (e.g., sham coil) to minimise potential placebo effects.

## Conclusion

Contrary to our hypotheses, one single session of prolonged iTBS with 80% RMT over LDLPFC and cerebellum did not enhance the behavioural and neurophysiological effects on working memory or cognitive control in healthy participants.

Equivalence testing was not performed to confirm whether iTBS at the DLPFC/cerebellum was statistically similar to sham. However, inspection of effect sizes and their 95% confidence intervals (see supplementary materials Fig. S1) suggested that there were neither cognitive nor ERP effects following a single stimulation session administered to both regions.

### Supplementary Information

Supplementary Information.

## Data Availability

MATLAB scripts used for EEG processing are available at the following link: https://github.com/EchoXu9/LDPFC-Cerebellum-iTBS.
