# Supplementary material for: Prolonged intermittent theta burst stimulation targeting the left prefrontal cortex and cerebellum does not affect executive functions in healthy individuals
Source: Sci Rep. 2024 May 24;14:11847. doi: 10.1038/s41598-024-61404-9 (PMC11116424; doi:10.1038/s41598-024-61404-9)
Supplement: Supplementary file 1 — Supplementary Information. [file 41598_2024_61404_MOESM1_ESM.pdf]

Supplementary Materials

Prolonged Intermittent Theta Burst Stimulation Targeting the Left Prefrontal Cortex and Cerebellum Does Not Affect Executive Functions in Healthy Individuals

Mei Xu[1], Stevan Nikolin[1, 2], Adriano H. D. M. Moffa[1, 2], Xiao Min Xu[1], Yon Su[1], Roger Li[1], Ho Fung Chan[1], Colleen K. Loo[1, 2, 3], Donel M. Martin[1, 2*]

[1]Discipline of Psychiatry and Mental Health, School of Clinical Medicine, Faculty of Medicine and Health, University of New South Wales, Sydney, Australia

[2]Black Dog Institute, Sydney, Australia

[3]The George Institute for Global Health, Sydney, Australia

***Correspondence**:

Donel M. Martin

Email: donel.martin@unsw.edu.au

Address: UNSW Sydney, High St, Kensington, NSW 2052, Australia

Table S1 Summary of results for ERPs amplitudes in 2-back and Stroop

Condition	Estimate	SE	z-value	*d*	95% CI LB	95% CI UB	*p*-value
2-back P300							
Sham	5.06	0.79	6.43	-	3.53	6.59	-
Cerebellum	5.17	0.81	6.36	0.02	3.59	6.75	0.84
DLPFC	5.08	0.80	6.36	0.00	3.53	6.64	0.96
2-back N200							
Sham	-0.62	0.71	-0.87	-	-2.00	0.77	-
Cerebellum	-0.39	0.74	-0.53	0.05	-1.82	1.04	0.65
DLPFC	-0.29	0.72	-0.40	0.09	-1.70	1.12	0.51
Stroop N200 congruent trials							
Sham	-0.64	0.51	-1.24	-	-1.63	0.36	-
Cerebellum	-0.88	0.52	-1.69	-0.14	-1.90	0.14	0.42
DLPFC	-0.68	0.52	-1.31	-0.04	-1.69	0.33	0.89
Stroop N200 incongruent trials							
Sham	-0.42	0.52	-0.80	-	-1.44	0.60	-
Cerebellum	-0.65	0.54	-1.22	-0.13	-1.69	0.39	0.47
DLPFC	-0.30	0.53	-0.56	0.04	-1.34	0.74	0.69
Stroop N450 congruent trials							
Sham	1.47	0.52	2.84	-	0.46	2.47	-
Cerebellum	1.27	0.53	2.40	-0.10	0.24	2.29	0.52
DLPFC	1.31	0.53	2.49	-0.07	0.29	2.33	0.61
Stroop N450 incongruent trials							
Sham	1.23	0.56	2.19	-	0.13	2.32	-
Cerebellum	1.22	0.57	2.13	-0.01	0.10	2.33	0.98
DLPFC	1.19	0.57	2.08	-0.03	0.08	2.30	0.91

Note: SE = standard error, CI = confidence interval, LB = lower bound, UB = upper bound, RT = reaction time, DLPFC = Dorsolateral prefrontal cortex. For DLPFC and cerebellar stimulations, *p*-values were computed compared to sham. Two-tailed tests and a significance level with *p*-value < 0.05 were used.

Table S2 Percentage of correct responses in the Stroop task

	Mean (%)	SD (%)	Median (%)	Min (%)	Max (%)	Q1 (%)	Q3 (%)
Sham	95.7	3.9	96.8	74.4	100	94.2	98.1
LDLPFC	95.5	4.8	96.8	73.1	100	94.2	98.7
Cerebellum	95.3	4.6	96.8	75.0	100	94.2	98.1

Note: SD = standard deviation, Q1 = first quartile, Q3 = third quartile.

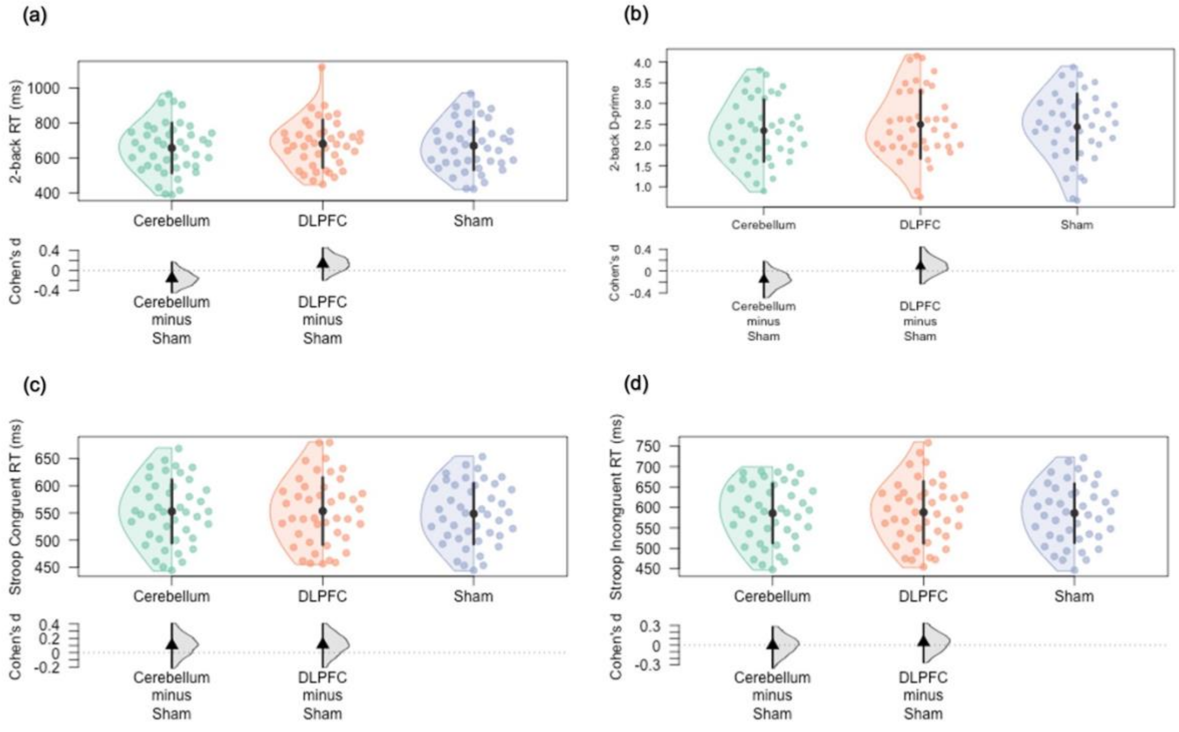

Fig. S1. Effect sizes (Cohen's d) of behavioural outcomes in 2-back and Stroop tasks. In scatter plots, black dots indicate the mean and black lines show the standard deviation. In effect sizes plots, black triangles reflect the mean bootstrapped effect size and grey regions indicate the 95% confidence interval. Effect sizes were computed from active conditions (i.e., LDLPFC and cerebellar prolonged iTBS) compared to sham. The effect sizes from both 2-back working memory and Stroop tasks were relatively small ranging from -0.4 to 0.4. **(a)** Scatter and effect size plots of reaction times in the 2-back. **(b)** Scatter and effect size plots of d-prime in the 2-back. **(c)** Scatter and effect sizes plots of reaction times for Stroop congruent trials. **(d)** Scatter and effect sizes plots of reaction times for Stroop incongruent trials.
